# Inferring biochemical reaction pathways: the case of the gemcitabine pharmacokinetics

**DOI:** 10.1186/1752-0509-6-51

**Published:** 2012-05-28

**Authors:** Paola Lecca, Daniele Morpurgo, Gianluca Fantaccini, Alessandro Casagrande, Corrado Priami

**Affiliations:** 1 The Microsoft Research - University of Trento Centre for Computational and Systems Biology, , 38068 Rovereto, Italy; 2 Department of Information Engineering and Computer Science - University of Trento, , Trento, Italy

## Abstract

**Background:**

The representation of a biochemical system as a network is the precursor of any mathematical model of the processes driving the dynamics of that system. Pharmacokinetics uses mathematical models to describe the interactions between drug, and drug metabolites and targets and through the simulation of these models predicts drug levels and/or dynamic behaviors of drug entities in the body. Therefore, the development of computational techniques for inferring the interaction network of the drug entities and its kinetic parameters from observational data is raising great interest in the scientific community of pharmacologists. In fact, the network inference is a set of mathematical procedures deducing the structure of a model from the experimental data associated to the nodes of the network of interactions. In this paper, we deal with the inference of a pharmacokinetic network from the concentrations of the drug and its metabolites observed at discrete time points.

**Results:**

The method of network inference presented in this paper is inspired by the theory of time-lagged correlation inference with regard to the deduction of the interaction network, and on a maximum likelihood approach with regard to the estimation of the kinetic parameters of the network. Both network inference and parameter estimation have been designed specifically to identify systems of biotransformations, at the biochemical level, from noisy time-resolved experimental data. We use our inference method to deduce the metabolic pathway of the gemcitabine. The inputs to our inference algorithm are the experimental time series of the concentration of gemcitabine and its metabolites. The output is the set of reactions of the metabolic network of the gemcitabine.

**Conclusions:**

Time-lagged correlation based inference pairs up to a probabilistic model of parameter inference from metabolites time series allows the identification of the microscopic pharmacokinetics and pharmacodynamics of a drug with a minimal a priori knowledge. In fact, the inference model presented in this paper is completely unsupervised. It takes as input the time series of the concetrations of the parent drug and its metabolites. The method, applied to the case study of the gemcitabine pharmacokinetics, shows good accuracy and sensitivity.

## Background

Drug metabolism is a process by which pharmaceutical substances undergo biochemical modification in living organisms. Such modifications may lead to the generation of an active drug from an inactive precursor or in other cases lead to the generation of an inactive compound from an active substance, aiding in detoxication. This process is performed by a network of biochemical reactions that modify the pharmaceutical substances. The mechanisms of action of a drug refer to the specific biochemical, chemical, or physical interactions through which a drug substance exerts its pharmacological effect. The biochemical transformations of a drug form part of the pharmacokinetic pathway, whereas the interactions of the drug on its targets compose the pharmacodynamic pathway. Our study focuses on the reconstruction of network of biochemical interactions taking place between the drug, its metabolites, and their cellular targets. In brief, reconstructing a pharmacokinetic model starting from in-vitro experiments.

At the macroscopic level, the pharmacokinetics concerns with the intracellular uptake kinetics and the metabolic transformations of a drug, usually performed by specialized enzymatic systems. Similarly, at the microscopic level, the pharmacodynamics concerns with biochemical interactions between the drug, or in some cases its metabolites, and their molecular targets. The drug metabolites and the drug targets are components of a network of interactions. This network is usually represented by a graph whose nodes are the system’s components (drug, metabolites and targets) and the edges between nodes link two biochemically interacting components. The directions of the edges specify the directions of the chemical reactions and the sign of the edges denotes the inhibitory or activatory effect of one component on another.

To infer the directional signed edges of the network it is common to use experimental time series of the concentration of the components of the systems, i.e. of the nodes of the network we want to infer. The network inferred from the species dynamics will include edges between those nodes whose time series exhibit sufficient functional connectivity, typically defined as a measure of coupling exceeding a predetermined threshold [1]. The same time series used to infer the network can be used also to estimate the rate constants that parametrize the interactions between the biochemical species. The preliminary network can be fitted to the experimental time series to eventually detect those edges whose kinetics is null, and that can be removed from the network.

In accordance to several studies of the late 1990s that indicated that poor understanding of pharmacokinetics properties and toxicity were important causes of costly late-stage failures in drug development [2], it has become appreciated the ability to infer and calibrate pharmacokinetic models, whose usefulness is now widely recognized through all the process of drug design and clinical development [3].

Our study proposes a method to deduce both reactions pathways and kinetic parameters of biotransformations of drugs from time series data of measured concentrations of metabolites. Determining the pathways in chemical reaction networks from time series data has been an active area of research over a decade. A comprehensive review of available techniques can be found in [4-6]. Unlike in the most part of network inference techniques, in our approach a probabilistic model of parameter inference is part of the procedure of the identification of interconnection topology. The integration of parameters estimation into a method of topology identification results in a well performing network inference algorithm. The parameter estimation works as a sort of model discriminator selecting only the interaction responsible for the kinetic of the system. In this way, the whole procedure is able to recover a good approximation of the true network topology, especially for metabolic networks. In this study, we focus on gemcitabine metabolic pathway. Gemcitabine is an oncological drug used in various carcinomas: non-small cell lung cancer, pancreatic cancer, bladder cancer and breast cancer [7]. It is being investigated for use in esophageal cancer, and is used experimentally in lymphomas and various other tumor types. The gemcitabine reaction pathway has been recently investigated [7] and new observational data provided by the experiments of Veltkamp et al. [8] contribute to elucidate the cellular pharmacology of gemcitabine. Therefore, the gemcitabine pathway is a good case study for our inference algorithm.

The majority of the literature about application of inference techniques in pharmacokinetics and pharmacodynamics modelling concerns the parameter estimation, rather then the identification of the interaction network structure. Moreover, the majority of the methods of parameter estimation fits continuous pharmacokinetics models to discrete time measurements of the drug’s kinetic and/or dynamic response. In pharmacokinetics, the first methods of inference were applied almost thirty years ago to the clinical problem of dose regimen [9]. They were mostly Bayesian methods.

More recently other parameter inference techniques and parameter fitting procedures have been developed. As a result, many software tools implementing them were developed and provided to the scientific community of modelers and pharmacologists (e.g. maximum likelihood inference techniques [10], parameter fitting by nonlinear mixed [11-13] effects are only two recent examples). Now, the application of inference and fitting procedures for parameter estimation has been extended to all phases of drug development [14], and it is no longer limited to the dose regime scheduling. In pharmacokinetics and pharmacodynamics, the parameter estimation techniques are applied to infer or fit two categories of parameters. The first category includes parameters for which a priori information is available, e. g. organ blood flows, organ volume fractions, receptor density, cellular signalling protein turnover, whereas the second category includes parameters with no a priori information, e. g. intrinsic clearance, transport and binding coefficients, or parameters representing specific properties of the drug such as partition coefficient.

The development of inference methods that deduce from time discretely observed concentrations of metabolites, both the network of the biotransformations and mechanisms of action as well as the kinetic parameters of these interactions is a new challenge of a new paradigm in drug design and discovery: the *network pharmacology* introduced by A. L. Hopkins [15]. Network pharmacology is grounded on *network biology*, that conceives a biological systems as a *network* whose nodes are the system’s components and the edges between nodes indicate the occurrence of interactions between the nodes. In the network-centric view of a biological system, the time evolution of biological systems, entities and processes is the result of the occurrence of the interactions among the system’s components. The dynamics of the network is governed by the kinetic parameters of the interactions. In most of the cases these parameters are not directly measurable and a priori knowledge is unavailable. Often, subjective prior beliefs on the parameters are difficult to be converted into a mathematically formulated model and prior. All these considerations motivated our work and our attempt to develop inference methods which are robust against noise, efficient in computation and flexible enough to meet different constraints.

**Figure 1:**
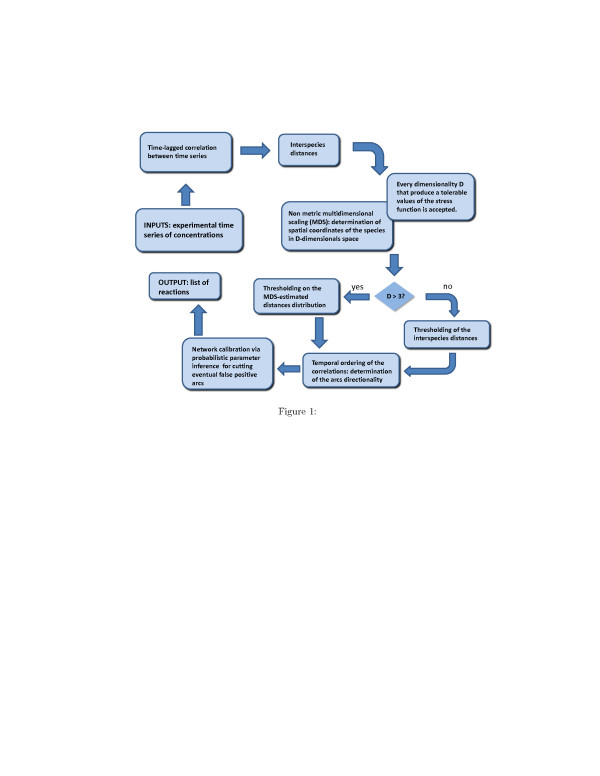
Scheme of the main steps of the network identification method.

The network inference method we propose is inspired to the recent works of M. Samoilov et al. [16] and A. Arkin et al. [17] and has been tailored for pharamcokinetics-pharmacodynamic modelling. It takes as an input the time series data of the concentrations of the parent drug and its metabolites. A prediction of the reaction network is deduced from time-lagged correlation functions of two chemical species at a time, obtained from concentration measurements. These functions are converted into interspecies distances whose analysis and visualization on a spatial domain yields the reaction pathway of the reacting system. To calibrate the network, i.e. to estimate the parameters, we developed an innovative probabilistic model of inference of the rate coefficients [18]. The tool implementing the parameter inference of a model of biochemical network, is the software KInfer [18]. KInfer is a software (free for non-commercial purposes) available for download from the software web page [19]. The only inputs required by KInfer are the list of chemical equations, or alternatively a generalized mass action, and the experimentally measured time-series of the reagents that are known to be involved in the system. Principal features of the tool are: automatic generation of generalized mass action model from the chemical reactions involved in the system; automatic estimation of the initial guesses and bounds for the parameter values; estimate of the propagation of the experimental errors from the input data to the parameter estimates; estimation of the level of noise in the input data. No a priori knowledge about parameter distribution is required by KInfer. A schematic overview of the main steps of the network identification method presented in this paper is shown in Figure 1. All the steps will be described in detail in the rest of the paper.

The paper outlines as follows: (i) we first present the model of inference based on the estimation of the time-lagged correlation functions; (ii) we review the parameter inference methods of KInfer; (iii) we describe the in vitro cytotoxicity, cellular uptake, efflux, biotransformation, and nucleic acid incorporation of the gemcitabine and present the experimental data used in this study; (iv) we show and discuss the inferred network of biotransformations and mechanisms of action of gemcitabine as obtained by our inference algorithm. Finally, (v) we end up the paper with some final conclusions and plans of future works.

## A time-lagged correlation based network inference

The time-lagged correlation is a measure that is related to the Pearson correlation coefficient but that takes into account shifts in time, i.e. lags, between the expression of the causal effector and the target module. The key-idea of correlation-based methods for network inference is that for a data set comprising time series profiles of *N* species, *X*_*i*_(*t*) for *i* = 1,…,*N*, the correlation matrix of the *N*(*N* − 1)/2 independent pairwise correlation coefficients can be used to cluster the data set into groups of species within which correlations between species are high, when compared to pairwise correlations between different groups. These groupings can most easily be discerned by calculating a matrix of pairwise *distances*, *d*_*ij*_ from the correlation matrix, whereby *d*_*ij*_ = 0 for two species which are completely positively correlated and increases as the pairwise correlation coefficient decreases.

The distance matrix can subsequently be analyzed to find, and visualize, patterns of proximities between species. A large family of techniques for the analysis and visualization of proximity data coming from similarity/distance matrix is available in literature. Many of these techniques refer to methods of data clustering, and, more recently, to generalized methods of graph splatting such as layout algorithms and multidimensional scaling algorithms [20-24]. The purpose shared by all these techniques is to reveal and, possibly, visualize, patterns of similarities and topological structures underlying the data. In this study we use a multidimensional scaling method. Multidimensional scaling transforms a distance matrix into a set of coordinates such that the distances derived from these coordinates approximate as well as possible the original distances. This way, the multidimensional scaling techniques are used in information visualization for exploring similarities or dissimilarities in data. In fact, the visualization of the similarities/distances among data facilitates the interpretation and the analysis of complex network, and, for this reason, they are currently becoming popular in bioinformatics [17,25,26] in network inference [16,17] and network analysis problems. We will describe later in the paper the details of the multidimensional scaling we used for this study.

Very often in practical situations the influence of one species on another takes some finite amount of time to propagate through the network, and the ordering of responses to impulse stimuli reveals information about network connectivity. In particular, this will be evident in the time series if the time interval *Δt* between concentration measurements is smaller than the characteristic response timescales for the network. Two time series which have a low correlation coefficient may in fact be strongly correlated if a time lag is allowed between the data points for the two species. Therefore, according to Arkin et al. [17] and Samoilov et al. [16], the covariance function between *X*_*i*_ and *X*_*j*_ is

(1) $$\begin{array}{lll} C_{\mathrm{ij}}(t,\tau ) & =\int_{{\hat{X}}_{i}^{\min}}^{{\hat{X}}_{i}^{\max}}\int_{{\hat{X}}_{j}^{\min}}^{{\hat{X}}_{j}^{\max}}(X_{i}(t)\;-\;\langle X_{i}\rangle )(X_{j}(t\;+\;\tau )\;-\;\langle X_{j}\rangle )\; & \;\\ & \;\times p(X_{i}(t),X_{j}(t+\tau ))dX_{i}dX_{j}\; \end{array}$$

where ${\hat{X}}_{i}^{\min\;(\max)}=\min{\left(\max\right)}_{t_{k}}\{{\hat{X}}_{i}(t_{k}),\;k=1,\ldots ,m\}$ (here, $\hat{X_{i}}$ denotes the observed value of the concentration of species i-th); *p*(*X*_*i*_(*t*),*X*_*j*_(*t* + *τ*)) is the pair distribution function, corresponding to the density of points on a scatter plot of *X*_*i*_(*t*) and *X*_*j*_(*t* + *τ*), and *m* is the number of measurements. The pair distribution function gives the density of points in the rectangle *dX*_*i*_ × *dX*_*j*_ on the plot (*X*_*i*_*X*_*j*_). *τ* is a delay (*time lag*) introduced to detect correlations that otherwise could be non-detectable. The delay *τ* can be estimated in the following way. Consider

$$\langle \tau_{i}\rangle \approx \frac{1}{m}\overset{m}{\underset{k=1}{\sum }}{\left(\left|\frac{1}{X_{i}(t_{k})-X_{i}(t_{k-1})}\frac{dX_{i}}{\mathrm{dt}}{|}_{t=t_{k}}\right|\right)}^{-1}$$

where $\frac{dX_{i}}{\mathrm{dt}}{|}_{t=t_{k}}$ can be calculated with the Stineman procedure [27] on the curve interpolating experimental time-series of species *i* at time point *t*_*k*_. We assume that

(2) $$\tau \in \left[0,\tau_{\min}\right]$$

and

(3) $$\tau_{\min}=\left[\underset{i}{\min}\{\tau_{i}\}\right]$$

The values of *τ* in (2) range over the interval of the rate limitingness across the entire reaction network.

Now, since generally the analytical expression of *p*(*X*_*i*_(*t*),*X*_*j*_(*t* + *τ*)) cannot be obtained, the calculation of the integral in Eq. (1) can be performed only switching from a continuous to a discrete domain, so that the integral can be approximated by a sum

(4) $$\begin{array}{lll} C_{\text{ij}}(t_{k},\tau ) & =\underset{\mu }{\sum }\underset{\nu }{\sum }\left\{(X_{i}^{\left(\mu ,\nu \right)}\left(t_{k}\right)-\langle X_{i}\rangle )(X_{j}^{\left(\mu ,\nu \right)}\left(t_{k}+\tau \right)\right)\; & \;\\ & \;-\langle X_{j}\rangle )p_{\mu \nu }\}.\; \end{array}$$

and, taking a time average over all of the measurements, we obtain a covariance matrix depending only on *τ*, as follows

(5) $$\begin{array}{lll} C_{\mathrm{ij}}(\tau ) & =\langle \underset{\mu }{\sum }\underset{\nu }{\sum }\left\{\left(X_{i}^{\left(\mu ,\nu \right)}(t_{k})-\langle X_{i}\rangle )(X_{j}^{\left(\mu ,\nu \right)}(t_{k}+\tau \right)\right)\; & \;\\ & \;-\langle X_{j}\rangle )p_{\mu \nu }\}\rangle .\; \end{array}$$

In order to estimate the pair distribution function *p*_*μν*_, Samoilov et al. [16] proposed to divide the space of the phase plane into rectangles of varying size so that the distribution of points is uniform in each rectangle. The algorithm developed by A. Fraser [28] is the most used procedure to perform such a partition of the phase plane *X*_*i*_*X*_*j*_. The pair distribution density can then be estimated as

(6) $$p_{\mu \nu }=\frac{N_{\mu \nu }}{N_{\mathrm{tot}}A_{\mu \nu }}$$

where *N*_*μν*_ is the number of points in the particular rectangle labeled *μν*, *N*_*tot*_ is the total number of points and *A*_*μν*_ is the area of the rectangle.

We propose here a different solution to the problem of the pair distribution function. Instead of diving the phase plane into rectangle of variable size, we propose a Voronoi tessellation of the space, following the results of the recent study of M. Browne [29] and Q. Du et al. [30]. This division of the space according to point proximity leads to region boundaries being straight lines, bisecting and running perpendicular to the line connecting the Delaunay neighbors. Boundary points are equidistant to exactly two sites, and vertices are equidistant to at least 3. Neighboring points are points whose associated Voronoi regions share a common boundary. Thus, Voronoi tessellation generates a clustering of the points in the phase plane that in a good approximation satisfies the requirement of homogeneity for the distribution of points inside a cell, and *p*_*μν*_ in Eq. (4) can be calculated as follows

(7) $$p_{\mu \nu }=1/\text{Area}(V_{\mu \nu })$$

where *V*_*μν*_ is the Voronoi cell *μν*.

Once, the covariance matrix has been calculated, the time-lagged correlation matrix **R**(*τ*) can be calculated according to the definition in Eq. (8) [16,17].

(8) $$r_{\mathrm{ij}}(\tau )=\frac{C_{\mathrm{ij}}(\tau )}{\sqrt{\left|C_{\mathrm{ii}}(\tau )C_{\mathrm{jj}}(\tau )\right|}}$$

Finally, from the correlation matrix we calculate a *distance matrix ***D** whose elements are defined in Eq. (9).

(9) $$d_{\mathrm{ij}}=\sqrt{\left|c_{\mathrm{ii}}-2c_{\mathrm{ij}}+c_{\mathrm{jj}}\right|}$$

where

(10) $$c_{\mathrm{ij}}=\underset{\tau }{\max}|r_{\mathrm{ij}}(\tau )|$$

is the maximum absolute value of the correlation between two species with a time lag *τ*.

The distances are used to find the connections between the different species in the system. Namely, the distances measure the relatedness of the time series describing the time behaviour of the species; the more related the more likely that two species are connected by a single reaction. The greater the distance between two species the more likely that two species are connected by several intermediate reactions or they are not connected at all.

Inspired by [16,17,31,32], we analyzed the distance matrix elements with a multidimensional scaling algorithm. A multi-dimensional scaling algorithm starts with a matrix of species-species distances. Then it assigns a location, i.e. the spatial coordinates to each item (species) in a D-dimensional space, where *D* is specified a priori. We used the Kruskal-Shepard multidimensional scaling [20]. This scaling is defined in terms of minimization of a cost function called *stress function* which is a measure of lack of fit between distances *d*_*ij*_ and distances ||*x*_*i*_−*x*_*j*_||. The stress is a residual sum of squares:

(11) $$S_{D}(x_{1},x_{2},\ldots ,x_{n})={\left[\underset{i\neq j}{\sum }{\left(d_{\mathrm{ij}}-||x_{i}-x_{j}||\right)}^{2}\right]}^{\frac{1}{2}}$$

so that Kruskal-Shepard scaling is also known as least-squares scaling [31]. This scaling seeks values of the coordinates $x_{1},x_{2},\ldots ,x_{n}\in R^{D}$ to minimize *S*. For a given value of *D* the estimation of the coordinates is performed in such a way that the pairwise distances are preserved as well as possible. The choice of *D* is arbitrary in principle, but low in practice: D = 2; 3 are the most frequently used dimensions, for the simple reason that the points serve as easily visualized representors of the species. A downhill simplex (amoeba) algorithm is used to minimize *S*_*D*_[33,34]. In the application domain of this study, the downhill simplex method turned out to be more efficient that the gradient descendant minimization method that is usually exploited in the Kruskal-Shepard scaling algorithm.

In this study we reported the 2D and the 3D visualizations of the networks. Nevertheless, a set of Euclidean distances on *p* points can be represented exactly (i.e. with *S*_*D*_=0) in at most *p*−1 dimensions. An insufficient number of dimensions is not the only cause of non zero stress. It may be caused also by random measurements errors in the input data. In such cases, even if the “true” number of dimensions of the problem were known, this would not guarantee that the stress corresponding to that number of dimensions is null [35-37]. Unfortunately, in most datasets the true dimensionality of the problem is not known in advance, like in the case treated in this study. The commonly advocated procedure for determining the dimensionality is a heuristic one of seeking a sharp drop or “elbow” in the rate of decline of stress as dimensionality increases. In practice such elbows are rarely obvious. In fact is has often noted that the pattern of change of stress versus dimensionality, rather than having an elbow, is characterized by a smooth and gradual decline [38]. As reported by M. D. Lee in [38], although there are at least two variants [39,40] of the multidimensional scaling that attempt to determine automatically the number of dimensions of the spatial representation they derive, there is not a rigorous and principled basis for this determination.

However, it not necessary that a multidimensional scaling representation has zero stress in order to be informative and useful. A certain amount of distortion is tolerable [37]. The amount of stress to tolerate can be derived from the accuracy of the input time series. The measurement error on the input data propagates to the distance matrix entries and, consequently, to the estimate of the stress function in the following way. For convenience, we introduce the following notation

$$\begin{array}{lll} & S_{i}(t)\equiv X_{i}(t)-{\bar{X}}_{i}\; & \;\\ & S_{j}(t+\tau )\equiv X_{j}(t+\tau )-{\bar{X}}_{j}\; & \;\\ & p(X_{i}(t),X_{j}(t+\tau ))\equiv p_{i}\;j(t,\tau ).\; & \; \end{array}$$

Then, we apply the rules of error propagation [41] to find the error on the covariance estimate given in Eq. 1.

The relative error on *C*_*i*_*j*(*t*,*τ*) is

(12) $$\begin{array}{lll} \frac{\delta C_{\mathrm{ij}}(\mathrm{t.\tau})}{C_{\mathrm{ij}}(\mathrm{t.\tau})} & \;=\;\int \;\int \;\sqrt{{\left(\;\frac{\delta S_{i}(t)}{S_{i}(t)}\;\right)}^{2}\;+\;{\left(\;\frac{\delta S_{j}(t\;+\;\tau )}{S_{j}(t\;+\;\tau )}\;\right)}^{2}\;+\;{\left(\;\frac{\delta p_{\mathrm{ij}}(t,\tau )}{p_{\mathrm{ij}}(t)}\;\right)}^{2}}\; & \;\\ & \;\;\times dX_{i}dX_{j}\; \end{array}$$

where

(13) $$\delta S_{i}(t)=\delta X_{i}(t)+\delta {\bar{X}}_{i}$$

(14) $$\delta S_{j}(t+\tau )=\delta X_{j}(t+\tau )+\delta {\bar{X}}_{j}$$

We assume that the estimate of the relative error of the density *p*_*ij*_(*t*,*τ*) is such that

$${\left(\frac{\delta p_{\mathrm{ij}}(t,\tau )}{p_{\mathrm{ij}}(t)}\right)}^{2}\ll {\left(\frac{\delta S_{i}(t)}{S_{i}(t)}\right)}^{2}+{\left(\frac{\delta S_{j}(t+\tau )}{S_{j}(t+\tau )}\right)}^{2}$$

so that, the absolute error on *C*_*ij*_(*t*,*τ*) is

(15) $$\begin{array}{lll} \delta C_{\mathrm{ij}}(t,\tau ) & =C_{\mathrm{ij}}(t,\tau )\cdot \;\int \;\int \;\sqrt{{\left(\;\frac{\delta S_{i}(t)}{S_{i}(t)}\;\right)}^{2}\;+\;{\left(\;\frac{\delta S_{j}(t\;+\;\tau )}{S_{j}(t\;+\;\tau )}\;\right)}^{2}}\; & \;\\ & \;\times dX_{i}dX_{j}\; \end{array}$$

and the absolute error on the time average of *C*_*ij*_(*t*,*τ*) is

(16) $$\delta C_{\mathrm{ij}}(\tau )=\frac{1}{m}\overset{m}{\underset{k=1}{\sum }}\delta C_{\mathrm{ij}}(t_{k},\tau )$$

where *m* is the number of records in the time series.

From the Eq. (8), the absolute error on the correlation coefficient *r*_*ij*_ is

(17) $$\delta r_{\mathrm{ij}}(\tau )\;=\;r_{\mathrm{ij}}(\tau )\;\cdot \;\sqrt{\;{\left(\;\frac{\delta C_{\mathrm{ij}}(\tau )}{C_{\mathrm{ij}(\tau )}}\;\right)}^{2}\;+\;\frac{1}{2}\left[\;{\left(\;\frac{\delta C_{\mathrm{ii}}(\tau )}{C_{\mathrm{ii}}(\tau )}\;\right)}^{2}\;+\;{\left(\;\frac{\delta C_{\mathrm{jj}}(\tau )}{C_{\mathrm{jj}}(\tau )}\;\right)}^{2}\;\right].}$$

Now, applying the rules of error propagation to the Eq. (9), we obtain that the absolute error on the distance *d*_*ij*_ is

(18) $$\delta d_{\mathrm{ij}}=\frac{1}{2}d_{\mathrm{ij}}\cdot \frac{\delta c_{\mathrm{ij}}+2\delta c_{\mathrm{ij}}+\delta c_{\mathrm{jj}}}{\left|c_{\mathrm{ii}}-2c_{\mathrm{ij}}+c_{\mathrm{jj}}\right|}=\frac{1}{2}d_{\mathrm{ij}}\cdot \frac{\delta r_{\mathrm{ij}}^{*}+2\delta r_{\mathrm{ij}}^{*}+\delta r_{\mathrm{jj}}^{*}}{\left|r_{\mathrm{ii}}^{*}-2c_{\mathrm{ij}}+c_{\mathrm{jj}}\right|}$$

where, as per the definition in Eq. (10)

$$\delta r_{\mathrm{ij}}^{*}=\underset{\tau }{\max}|\delta r_{\mathrm{ij}}(\tau )|$$

with *δr*_*ij*_(*τ*) as in Eq. (17).

Therefore, in a given dimensionality *D* we can consider tolerable a non-zero value of the stress function if

(19) $$||z_{i}-z_{j}||\in \left[d_{\mathrm{ij}}-\delta d_{\mathrm{ij}},d_{\mathrm{ij}}+\delta d_{\mathrm{ij}}\right],\;\forall i,j$$

This condition can be satisfied for more than one non-null value of the dimensionality *D*. If the condition is satisfied for *D*=2,3, we will use these values as input to the multidimensional scaling procedure and the network can be visualized. In particular, *D*=3 is selected if, moving from two to three dimensions, we have a significant reduction in stress, i.e. if

(20) $$S_{D=3}+\delta S_{D=3}\leq S_{D=2}+\delta S_{D=2}$$

where *δS*_*D*_ is the error affecting the value of the stress function because of the error *δd*_*ij*_ on the distance *d*_*ij*_. *δS*_*D*_ is given as in the following

$$\delta S_{D}=2S_{D}\cdot \frac{\underset{i\neq j}{\sum }{\left(d_{\mathrm{ij}}-||z_{i}-z_{j}||\right)}^{2}\cdot \frac{\delta d_{\mathrm{ij}}}{d_{\mathrm{ij}}-||z_{i}-z_{j}||}}{\underset{i\neq j}{\sum }{\left(d_{\mathrm{ij}}-||z_{i}-z_{j}||\right)}^{2}}$$

**Figure 2:**
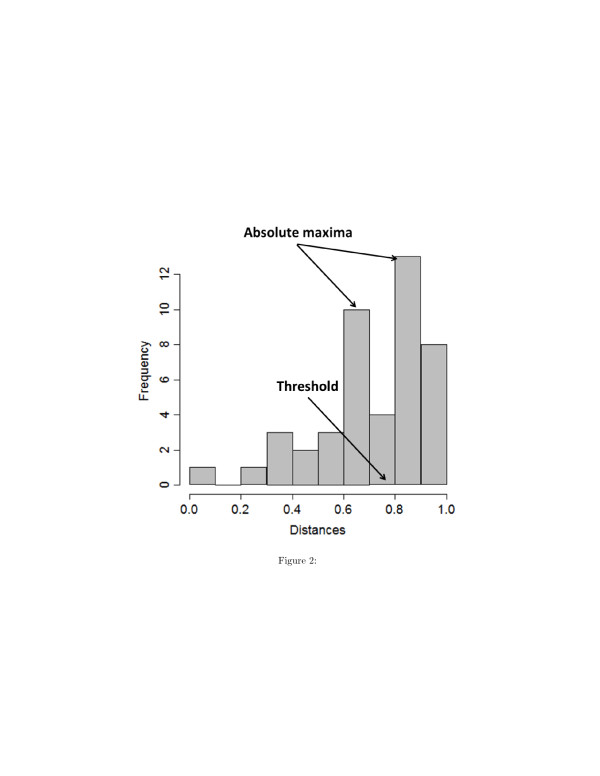
**Example of histogram of distances between chemical species.** It is used to determine a threshold under which an edge is drawn between two species. In this figure, the value of the threshold is 0.75.

If *D*≤3, once the coordinates of the species in *D*-dimensional space have been calculated from the distance matrix, we fix a threshold on the multidimensional-scaling estimated distances to establish when two species interacts through a single reactions or not. The threshold is calculated from the histogram of the distances, and it is set equal to the average of the values on the x-axis corresponding to the absolute maxima of the histogram (see Figure 2). If *D*>3, the multidimensional scaling procedure can be skipped and the original distance matrix can be directly analyzed and thresholded.

Then, we derive the undirected graph representing the network: species whose distance do not overcome the threshold undergo a biochemical interaction. In order to determine directions of the edge we need to infer a temporal ordering of the reaction events. Having knowledge of the temporal sequence of the reaction means knowing whether a perturbation of one species follows or proceeds that of another species. As suggested by Arkin et al. [17] the temporal ordering of variation in each of the variables can be assigned in the following way. If the time series for a given species has a maximum correlation at negative lags compared to a reference time series, then that species receives the input signals after the reference series, and vice versa. Similarly, if the two series are maximally correlated at zero lag but correlation tails to negative (positive) lags, variation in the given species closely follows (precedes) variation in the reference species. In this study, we do not consider negative lags, but without any loss of generality, we consider only positive lags and look for cause-effect relationships on a positive temporal scale.

In the next section we will describe the procedure of inference of the kinetic parameters of the interactions among the nodes of the network.

## Inferring the kinetic parameters with KInfer

The tool we recently developed to infer the kinetic parameters is KInfer (Kinetics Inference). It takes as an input the experimental time series of each reactants in the systems and a set of chemical reactions that are supposed to occur among the species of that system. Therefore, we apply KInfer to the network model we inferred with the time-lagged correlation based inference procedure described in the previous section.

We report here the key statements of the theory implemented by the tool and refer the reader to the references [18,42,43].

Consider *N* reactant species, with concentrations *X*_1_,*X*_2_,…,*X*_*N*_, that evolve according to a system of rate equations established by the generalized mass action law.

(21) $$\frac{dX_{i}}{\mathrm{dt}}=f_{i}(X^{\left(i\right)}(t);\theta_{i})=\overset{N_{i}}{\underset{h=1}{\sum }}\left(\theta_{\mathrm{ih}}\underset{w\in S_{h}}{\prod }X_{w}^{\alpha_{w}}\right)$$

where *θ*_*i*_ , *i*=1,2,…,*N* , is the vector of the rate coefficients, which are present in the expression of the function *f*_*i*_; *α*_*w*_∈**R**, and *N*_*i*_ is the number of parameter in the *f*_*i*_ rate equation.

We wish to estimate the set of parameters Θ={*θ*_*i*_} (*i*=1,2,…,*N*), whose element *θ*_*i*_ is the set of rate coefficients appearing in the rate equations of i-th species, therefore

$$\theta_{1}=\{\theta_{11},\theta_{12},\ldots ,\theta_{1N_{1}}\},\ldots ,\theta_{N}=\{\theta_{N1},\theta_{N2},\ldots ,\theta_{NN_{N}}\}$$

**X**^(*i*)^ is the vector of concentrations of chemicals that are present in the expression of the function *f*_*i*_ for the species *i*. We assume we have noisy observations $\hat{X_{i}}=X_{i}+\varepsilon$ at times *t*_0_,…,*t*_*M*_ , where $\varepsilon ∼N(0,\sigma^{2})$ is a Gaussian noise term with mean zero and variance *σ*. With this choice we are assuming that the concentration measurements are not significantly affected by systematic errors, but by uncontrolled random errors and that an error is equally likely to occur in either positive or negative direction with respect to the symmetry axis of the distribution. We also assume a number *M* of concentration measurements for each considered species. Approximating the rate Equation (21) as a finite difference equation between the observation times, gives

(22) $$X_{i}(t_{k})=X_{i}(t_{k-1})+(t_{k}-t_{k-1})f_{i}(X^{\left(i\right)}(t_{k-1});\theta_{i})$$

where *k*=1,…,*M*. In Eq. (22) the rate equation is viewed as a model of increments/decrements of reactant concentrations; i.e., given a value of the variables at time *t*_*k*−1_ , the model can be used to predict the value at the next time point *t*_*k*_. Increments/decrements between different time points are conditionally independent by the Markov nature of the model (22). Therefore, given the Gaussian model for the noise, the true value of *X*_*i*_(*t*_*k*_) is normally distributed around the observed value $\hat{X_{i}}(t_{k})$, so that

(23) $$\begin{array}{lll} p\left(X_{i}(t_{k-1})|{\hat{X}}_{i}(t_{k-1})\right) & =N\left({\hat{X}}_{i}(t_{k-1}),\sigma^{2}\right)=\frac{1}{\sqrt{2\Pi }\sigma }\; & \;\\ & \;\times \exp\left[-\frac{{\left(X_{i}(t_{k-1})-{\hat{X}}_{i}(t_{k-1})\right)}^{2}}{2\sigma^{2}}\right]\; \end{array}$$

Therefore, the probability to observe a variation *D*_*i*_(*t*_*k*_)=*X*_*i*_(*t*_*k*_)−*X*_*i*_(*t*_*k*−1_) for the concentration of the *i*-th species between the time *t*_*k*−1_ and *t*_*k*_, given the parameter vector *θ*_*i*_ is

(24) $$p(D_{i}(t_{k})|\theta_{i},\sigma )=N\left(E\left[f_{i}(X^{\left(i\right)}(t_{k-1}),\theta_{i})\right],2\sigma^{2}\right)$$

and

(25) $$\begin{array}{lll} E\left[f_{i}(X^{\left(i\right)}(t_{k-1},\theta_{i}))\right] & =\underset{\Omega_{X^{\left(i\right)}}}{\int }f_{i}(X^{\left(i\right)}(t_{k-1}),\theta_{i})\overset{K_{i}}{\underset{i=1}{\prod }}\; & \;\\ & \;\times \;\left[p_{i}\left(X_{i}(t_{k-1})|{\hat{X}}_{i}(t_{k-1})\right)\;\right]\;dX^{\left(i\right)}\; \end{array}$$

where $\Omega_{X^{\left(i\right)}}$ is the sample space of **X**^(*i*)^, and *K*_*i*_ is the number of chemical species in the expression for *f*_*i*_.

While the increments/decrements are conditionally independent given the starting point $X_{i}\left(t_{k}\right)$, the random variables *D*_*i*_(*t*_*k*_) are not independent of each other. Intuitively, if *X*_*i*_(*t*_*k*_) happens to be below its expected value because of random fluctuations, then the following increment *D*_*i*_(*t*_*k* + 1_) can be expected to be bigger as a result, while the previous one *D*_*i*_(*t*_*k*_) will be smaller. A simple calculation allows us to obtain the covariance matrix of the vector of increments for the *i*-th species. This is a banded matrix **C**_*i*_≡**C**=Cov(**D**_*i*_) with diagonal elements given by 2*σ*^2^ and a non-zero band above and below the diagonal given by −*σ*^2^. All other entries of **C** are null.

The likelihood for the observed increments/decrements therefore will be

(26) $$\begin{array}{lll} p(D|\Theta ) & =\overset{N}{\underset{i=1}{\prod }}N(D_{i}|m_{i}(\Theta ),C)={\left(\frac{1}{\sqrt{2\Pi \;\text{det}(C)}}\right)}^{N}\; & \;\\ & \;\times e^{\overset{N}{\underset{i=1}{\sum }}-\frac{1}{2}{\left(D_{i}-m_{i}\right)}^{T}C^{-1}(D_{i}-m_{i})}\; \end{array}$$

where **D**={**D**_1_,…,**D**_*N*_}, **D**_*i*_=*D*_*i*_(*t*_1_),*D*_*i*_(*t*_2_),…*D*_*i*_(*t*_*M*_) (*i*=1,2,…,*N*), and $m_{i}(t_{k-1})\equiv E\left[f_{i}(X(t_{k-1}),\theta_{i})\right]$.

Eq. (26) can be optimized w. r. t. the parameters Θ=(*θ*_1_,*θ*_2_,…,*θ*_*N*_) of the model to yield estimates of the parameters themselves and of the noise level. The inferred parameters are then used as multiplicative coefficients for each element of the correlation matrix, so that we obtain a weighted correlation matrix, reflecting the physical interaction in the network.

This procedure makes null the element of the correlation matrix if the rate constant multiplying this element is a null kinetic rate constant or if this rate constant is affected by a relative error of 50%.

## Metabolism and mechanisms of action of gemcitabine

Gemcitabine (2^*′*^,2^*′*^-difluorodeoxycytidine, dFdC) is an anticancer drug, which is effective against solid tumours, including non-small-cell lung cancer and pancreatic cancer. Gemcitabine is an anticancer nucleoside analog in which the hydrogen atoms on the 2^*′*^-carbon of deoxycytidine are replaced by fluorine atoms. As with fluorouracil and other analogues of pyrimidines, the triphosphate analogue of gemcitabine replaces one of the building blocks of nucleic acids, in this case cytidine, during DNA replication. The process arrests tumor growth, as only one additional nucleoside can be attached to the “faulty” nucleoside, resulting in termination of DNA replication and ultimately leading the cells to apoptosis.

**Figure 3:**
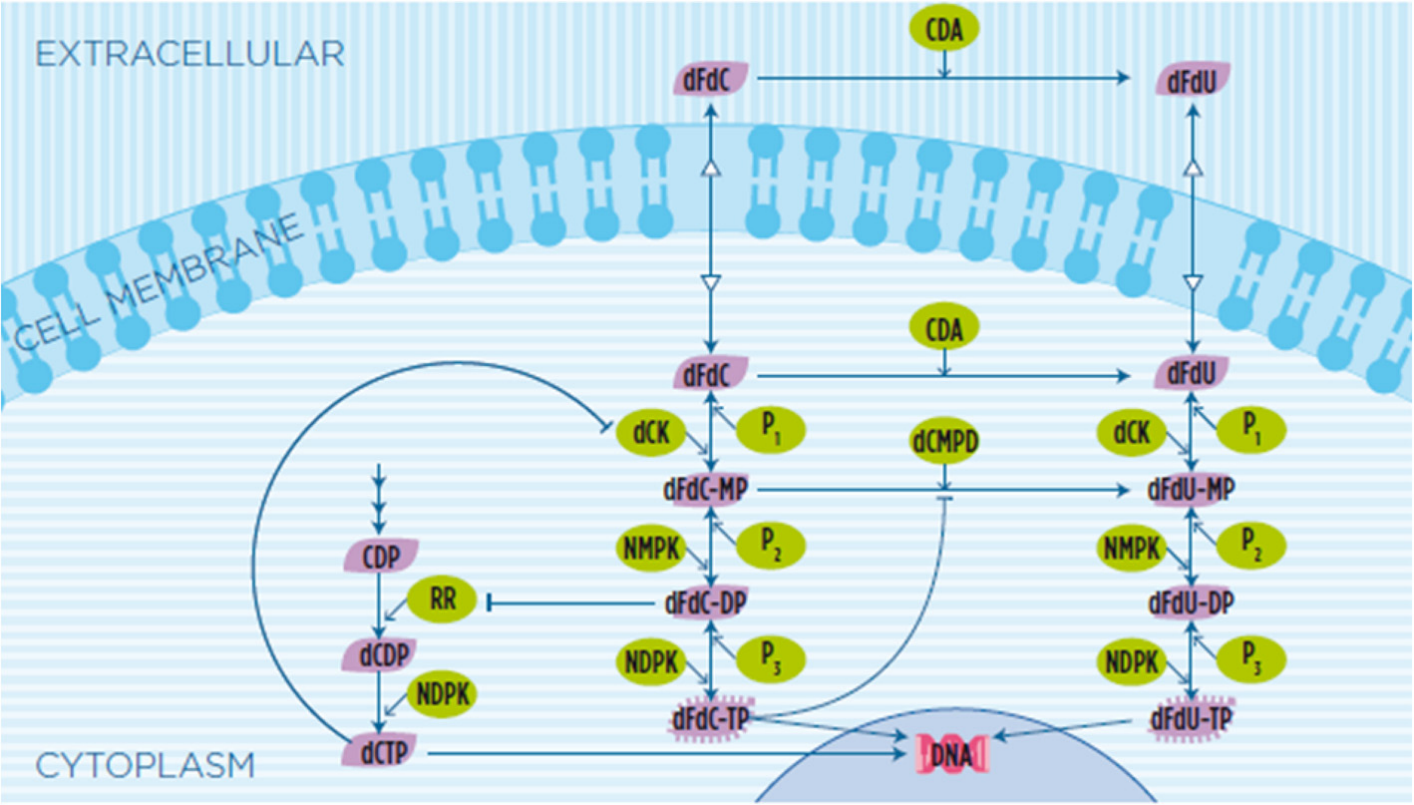
**Biotransformations and pharmacologic actions of dFdC and its metabolite as reported in a recent study of Veltkamp et al. [**[8]**].**

In this study we refer to the recent model proposed by Veltkamp et al. [8]. The model is depicted in Figure 3. The gemcitabine is transported into cells by equilibrative and concentrative nucleoside transporters. Then, it is phosphorylated by deoxycytidine kinase (dCK) to become the gemicitabine mono-phosphate (dFdC-MP). dFdC-MP is phosphorylated to its active diphosphorylated (dFdC-DP) and triphosphorylated (dFdC-TP) forms with the intervention of nucleoside monophosphate kinase (NMPK) and nucleoside diphosphate kinase (NDPK), respectively. The triphosphate metabolite (dFdC-TP) competes with the natural nucleoside triphosphate for the incorporation into the DNA and blocks cells in the early DNA synthesis phase. Gemcitabine is also rapidly metabolized by cytidine deaminase to 2^*′*^,2^*′*^-difluorodeoxyuridine (dFdU), which can be further phosphoriylated to its disphosphate (dFdU-DP) and triphosphate (dFdU-TP) whose activity has been recently associated with the cytotoxic effect of the drug [8].

**Figure 4:**
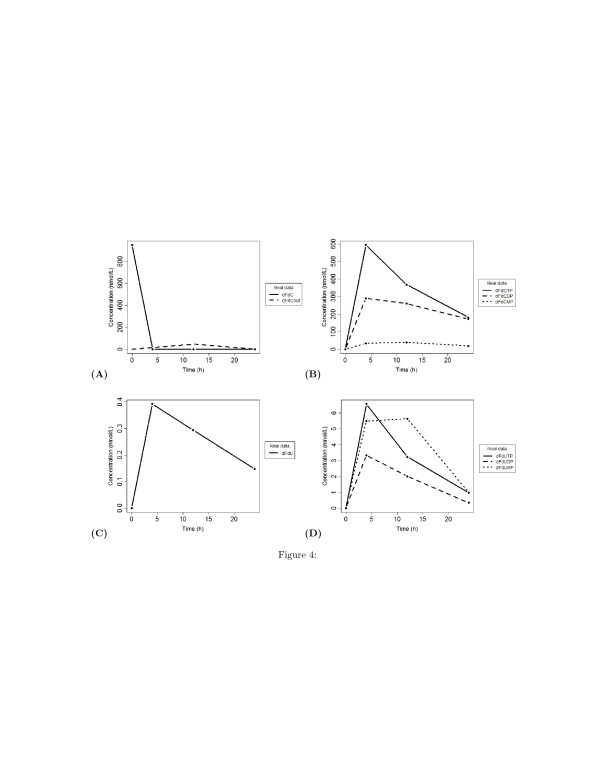
**Experimental concentration profiles of dFdC and dFdU metabolites [**[8]**].** Concentrations are expressed in nM/L and time is expressed in hours. **(A)** Time behaviour of extra-cellular and intra-cellular concentration of dFdC; **(B)** Time behaviour of phosphorylated metabolites of dFdC; **(C)** Time behavior of intra-cellular concentration of dFdU; **(D)** time behaviour of phosphorylated metabolites of dFdU.

In this study we used the time series data on the concentration of the gemcitabine and its metabolites published by Veltkamp et al. [8] and reported in Figure 4. The concentration of the following metabolites has been measured at four time points (0, 4, 12, and 24 hours): extracellular dFdC (dFdCout), intracellular dFdC, intracellular dFdC-MP, dFdC-DP, dFdC-TP, dFdU, dFdU-MP, dFdU-DP, and dFdU-TP.

## Results

We first applied the algorithm of network inference to deduce some of the biotransformations of gemcitabine from the experimental time series of metabolite concentrations available in [8]. The algorithm can infer the reactions between the measured species, therefore, since the experiments in [8] measured the concentrations of dFdCout, dFdC, dFdCMP, dFdCDP, dFdCTP, dFdU, dFdUMP, dFdUDP, dFdUTP, the reactions we expect to infer are only those among these chemical species.

**Figure 5:**
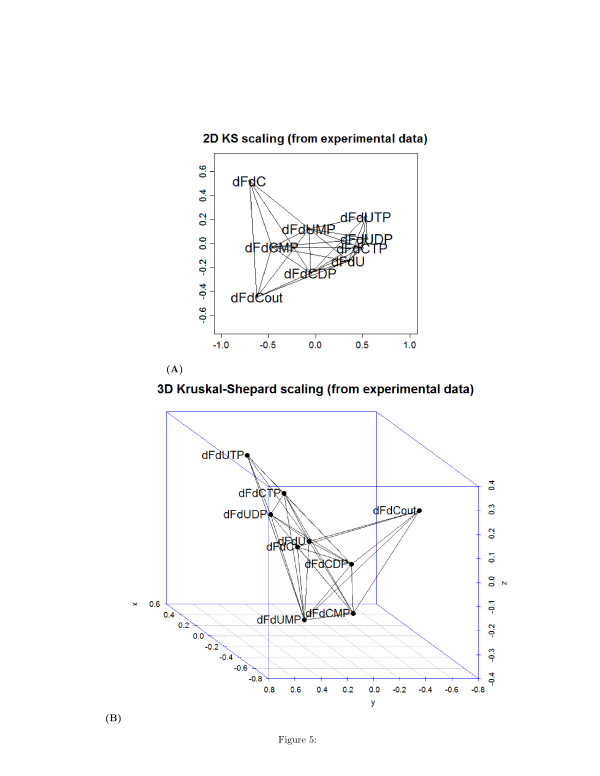
(A) Bi-dimensional and three-dimensional (B) wiring diagram obtained with a Kruskal-Shepard multidimensional scaling algorithm from real time series of metabolites concentration.

The Figures 5 (A) and 5 (B) show the undirected unsigned graph representing the network of interactions between the systems components, in a 2D and 3D space respectively The maximum value of the distance at which two species are still connected by an edge has been estimated to be 0.8. The interval ranged by *τ* has been calculated by the formulas (2) and (3) and is [0,6] hours.

**Figure 6:**
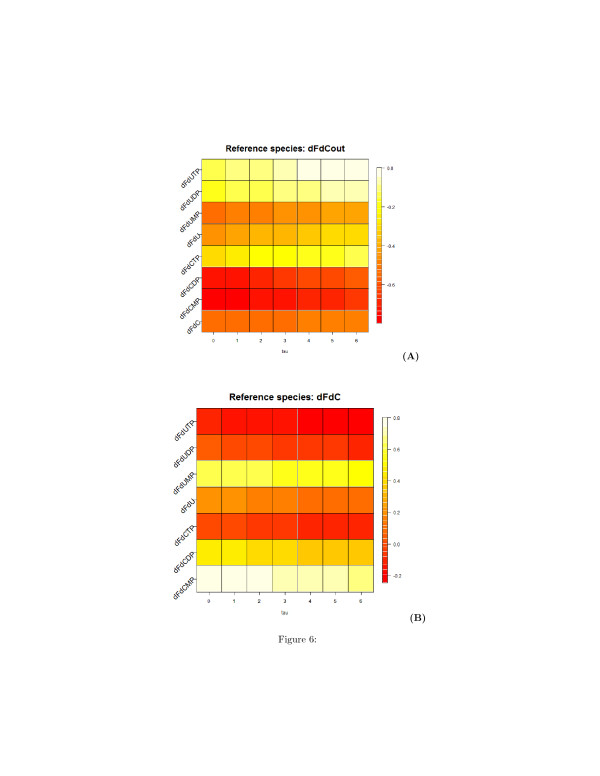
**Heatmap representation of the time-lagged correlations between species obtained from measured time series of metabolites concentration.(A)** Reference species is the extra-cellular dFdC; **(B)** Reference species is the intracellular dFdC. On the x-axis, the values of the delay *τ* are reported.

**Figure 7:**
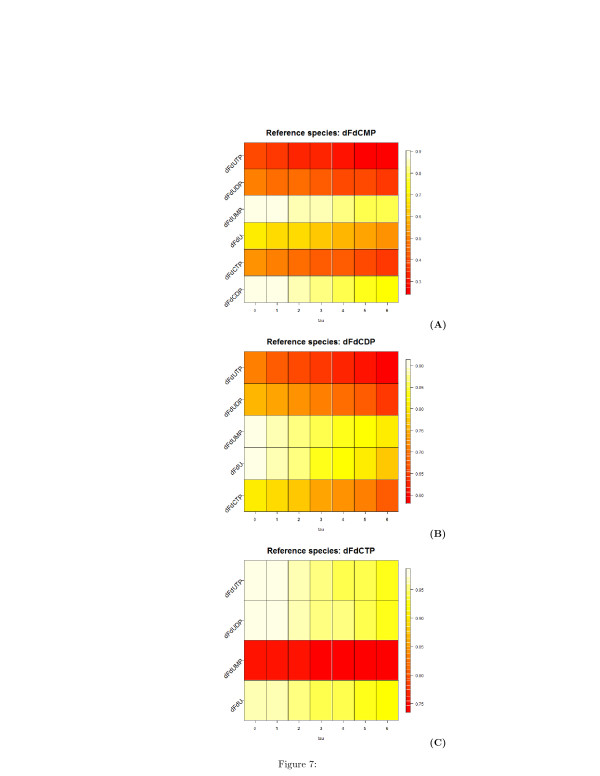
**Heatmap representation of the time-lagged correlations between species obtained from measured time series of metabolites concentration.(A)** Reference species is the mono-phosphate metabolite of dFdC. **(B)** Reference species is the di-phosphate metabolite of dFdC. **(C)** Reference species is the tri-phosphate metabolite of dFdC. On the x-axis, the values of the delay *τ* are reported.

**Figure 8:**
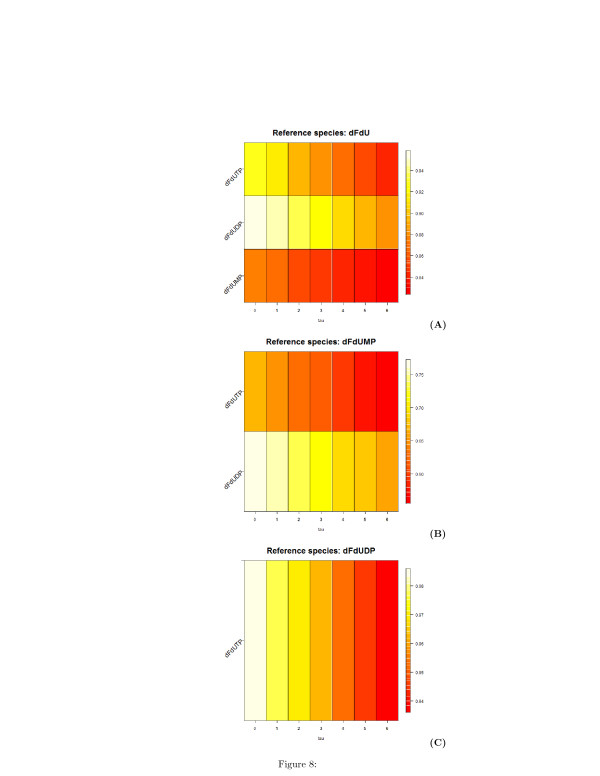
**Heatmap representation of the time-lagged correlations between species obtained from measured time series of metabolites concentration.(A)** Reference species is the intra-cellular of dFdU. **(B)** Reference species is the di-phosphate metabolite of dFdU. **(C)** Reference species is the di-phosphate metabolite of dFdU. On the x-axis, the values of the delay *τ* are reported.

The analysis of the pairwise time-lagged correlation **C**_*ij*_(*τ*) allows to deduce the directions of the edge of the graph. To facilitate the understanding of this analysis and its results, we visualized on a plot this correlation metric. Figures 6, 7, and 8 are heatmap representations of the behavior of the correlation **C**_*ij*_(*τ*) for each possible pairwise combination of the biochemical species. In Figure 6, the reference species are dFdCout (A) and dFdC (B), i.e. the extracellular and the intracellular gemcitabine, respectively. In Figure 7, the reference species are the mono- (A), di- (B) and tri-phosphate (C) of dFdC. In Figure 8, the reference species are dFdU (A), dFdUMP (B) and dFdUDP (C), i.e. the intracellular 2^*′*^,2^*′*^-difluorodeoxyuridine and its mono- and di-phosphate. A linear increment of the correlation is obtained in almost all the cases in the observed time interval. This behavior of the correlation indicates that most of the reactions are sequential and reversible. Species connected by a chain of sequential reversible reactions maintain a correlation significantly different from zero during all the observation time. Moreover, as we can see in Figures 4 (A) and 4 (B), these species appear to be connected by an edge if they do not directly interact. The linear increment of the correlation for almost all the couples of species makes not easy to dissect the chain of sequential reactions and distinguish between direct and indirect interactions. Table 1 reports the values of the lags corresponding to the maximum and minimum value of the correlation. In this table we see that the extra-cellular concentration of dFdC (dFdCout) is maximally anti-correlated to all the other species after 6 hours, whereas the minimal value of the correlation is obtained for *τ*=0. This results is consistent with the behavior of the experimental time series in Figure 4, and is explained by the time resolution of the measurements. Although the concentration of dFdCout rapidly decreases within the first 5 hours, the increment of the intracellular dFdC slowly increases and reaches the maximum around *t*=12 hours. Therefore the correlation between dFdCout and dFdC and between dFdCout and the phosphorilated metabolites of dFdC and dFdU is detected only after 6 hours (see the first part of Table 1). Conversely, the correlation between dFdCout and dFdC, as well as the correlation between dFdCout the phosphorilated metabolites of dFdC and dFdU reaches a minimum at *τ*=0 (see the first part of Table 1). Table 1 also shows that dFdC and its phosphorilated metabolites are maximally correlated at *τ*=0. dFdC is maximally correlated to the dFdU metabolites at *τ*=0 as well (see third part of Table 1). Similarly the correlation between the metabolites of dFdC and dFdU has a maximum at *τ*=0 (see parts 2, 3, 4, 5, 6, 7 of Table 1).

**Table 1:** **Minimum and maximum correlation and corresponding value of the lag*τ*(*τ***_**min_corr**_**and*τ***_**max_corr**_**, respectively) obtained from real experimental data**

**Reference species**	**Species**	***τ***_***max_corr***_	**Max correlation**	***τ***_***min_corr***_	**Min correlation**
dFdCout	dFdC	6	-0.5131595	0	-0.5282891
dFdCout	dFdCMP	6	-0.6701863	0	-0.7983718
dFdCout	dFdCDP	6	-0.574825	0	-0.7518225
dFdCout	dFdCTP	6	-0.1352853	0	-0.2802762
dFdCout	dFdU	6	-0.2821309	0	-0.4398082
dFdCout	dFdUMP	6	-0.4024222	0	-0.5185132
dFdCout	dFdUDP	6	-0.04846271	0	-0.1694
dFdCout	dFdUTP	6	0.001938335	0	-0.1202802
dFdC	dFdCMP	0	0.8020463	6	0.6897214
dFdC	dFdCDP	0	0.4703194	6	0.3333613
dFdC	dFdCTP	0	-0.01175614	6	-0.1341448
dFdC	dFdU	0	0.2034577	6	0.07041349
dFdC	dFdUMP	0	0.6428315	6	0.5227021
dFdC	dFdUDP	0	0.01913633	6	-0.09642542
dFdC	dFdUTP	0	-0.1380516	6	-0.2471314
dFdCMP	dFdCDP	0	0.900924	6	0.7347594
dFdCMP	dFdCTP	0	0.5163396	6	0.3631771
dFdCMP	dFdU	0	0.6947208	6	0.5328304
dFdCMP	dFdUMP	0	0.9040247	6	0.7781543
dFdCMP	dFdUDP	0	0.4910136	6	0.3534895
dFdCMP	dFdUTP	0	0.3758334	6	0.2393577
dFdCDP	dFdCTP	0	0.8094718	6	0.6765684
dFdCDP	dFdU	0	0.9141036	6	0.77697
dFdCDP	dFdUMP	0	0.9074831	6	0.8139078
dFdCDP	dFdUDP	0	0.7619494	6	0.6458895
dFdCDP	dFdUTP	0	0.6994546	6	0.580302
dFdCTP	dFdU	0	0.9740346	6	0.9208324
dFdCTP	dFdUMP	0	0.7554619	6	0.7344294
dFdCTP	dFdUDP	0	0.9872751	6	0.9356584
dFdCTP	dFdUTP	0	0.9856026	6	0.9283316
dFdU	dFdUMP	0	0.872246	6	0.8236066
dFdU	dFdUDP	0	0.9585976	6	0.8808236
dFdU	dFdUTP	0	0.9251018	6	0.8427518
dFdUMP	dFdUDP	0	0.7730816	6	0.6595772
dFdUMP	dFdUTP	0	0.6680645	6	0.5550562
dFdUDP	dFdUTP	0	0.9860277	6	0.936058
dFdUTP	dFdUTP	0	1	6	0.9634716

**Figure 9:**
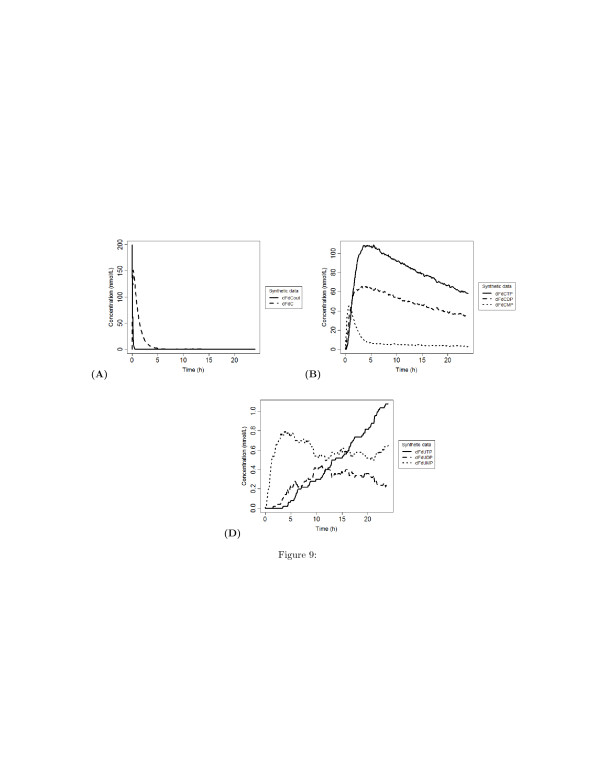
**Simulated time behaviour of parent drug and its metabolites.** The curves have ben obtained by simulating a mass action model of the interactions depicted in the cartoon of Figure 2. A level of noise equal to the 7% of the concentration values has been artificially introduced to simulate the presence of experimental uncertainties. In this model we asssumed that both the intra- and the intra-cellular concetration of dFdU reach the equilibrium within the first four hours. **(A)** Time behaviour of extra- and intra-cellular concentrations of dFdC. **(B)** Time behaviour of the concentrations of phisphorylated metabolites of dFdC. **(C)** Time behaviour of the concentrations of phisphorylated metabolites of dFdU.

Although these results are consistent with the dynamics measured in the experiments (see Figure 4), they do not reflect the expected kinetics in the first 4 hours. In fact, the kinetics of uptake of dFdC is expected to be faster than the one observed in the experiments. In particular, the rate of increment of the intracellular concentration of dFdC is expected to be proportional to the decrement of its extracellular concentration within the first 4 hours. We argued that the time resolution of measurements of the concentration of the metabolites does not allow to detect the dynamics of the concentration in the first 4 hours. As a consequence, the observed slow increment of dFdC in spite of the rapid decrement of its external concentration, and the observed peaks of the concentration of dFdC metabolites after 6 hours should be the reason why the maximum correlation between dFdCout and dFdC and its metabolites is reached at 6 hours. In order to demonstrate this guess, and to check the capability of our algorithm to correctly detect the connectivity between the biochemical species, we simulated a mass action model of the metabolism and mechanisms of action depicted by the cartoon in Figure 3. The aim of this experiment is to validate the capability of the inference procedure to correctly identify the simulated model, i.e. to detect all the interactions specified in the model. The model parameters have been obtained by fitting the model to the experimental time series of Veltkamp et al. [8]. The best-fitting parameters have been estimated with a downhill simplex technique [34] and provide the dynamics showed in Figure 9. Unlike the experimental observation, the simulation of time behaviour of the internal dFdC has a maximum within the first 4 hours (Figure 9 (A)). Although this maximum is expected, it has been not detected in the experiments because of the coarse-grain time resolution of the records.

We used the time series of dFdCout, dFdC, dFdCMP, dFdCDP, dFdCTP, dFdUout, dFdU, dFdUMP, dFdUDP, dFdUTP generated by the simulation of the model as input to the inference algorithm. The simulated time series contain 200 points regularly spaced in the interval [0, 20] hours (see the curves in Figure 9). Unlike the set of real data, this set contains also a time course of the extracellular concentration of dFdU (dFdUout).

**Figure 10:**
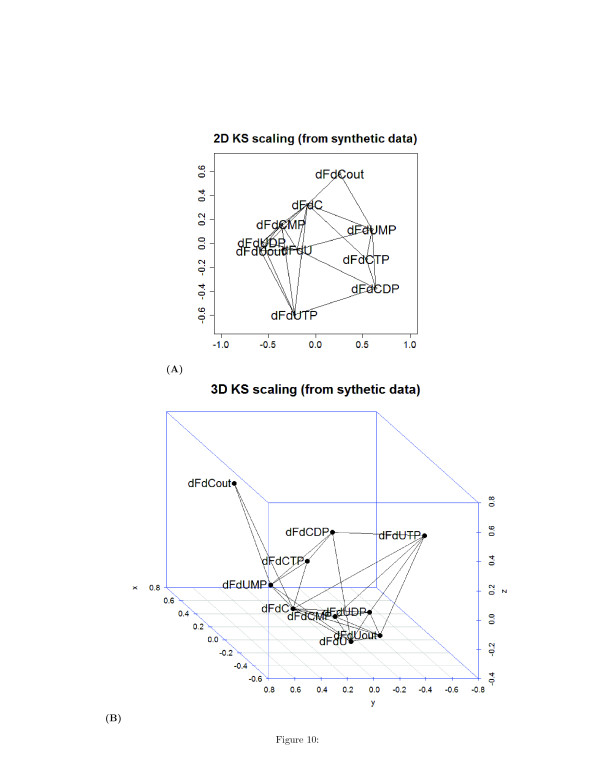
(A) Bi-dimensional and three-dimensional (B) wiring diagram obtained with a Kruskal-Shepard multidimensional scaling algorithm from synthetic time series of metabolites concentration.

**Figure 11:**
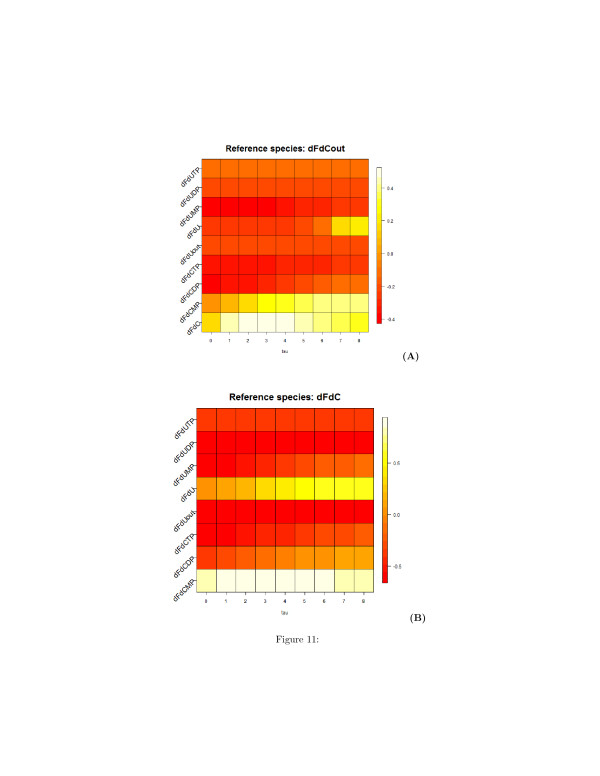
**Heatmap representation of the time-lagged correlations between species obtained from synthetic time series of metabolites concentration.(A)** Reference species is the extra-cellular dFdC; **(B)** Reference species is the intracellular dFdC. On the x-axis, the values of the delay *τ* are reported.

**Figure 12:**
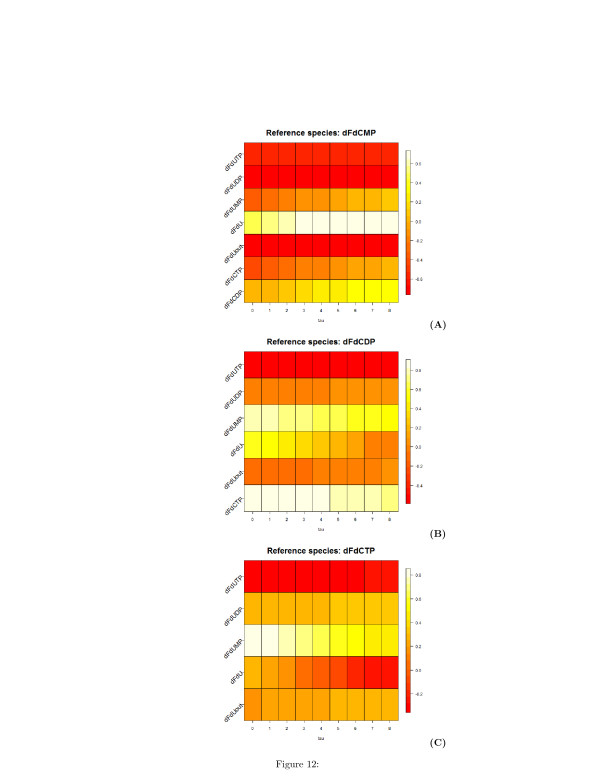
**Heatmap representation of the time-lagged correlations between species obtained from synthetic time series of metabolites concentration.(A)** Reference species is the mono-phosphate metabolite of dFdC. **(B)** Reference species is the di-phosphate metabolite of dFdC. **(C)** Reference species is the tri-phosphate metabolite of dFdC. On the x-axis, the values of the delay *τ* are reported.

**Figure 13:**
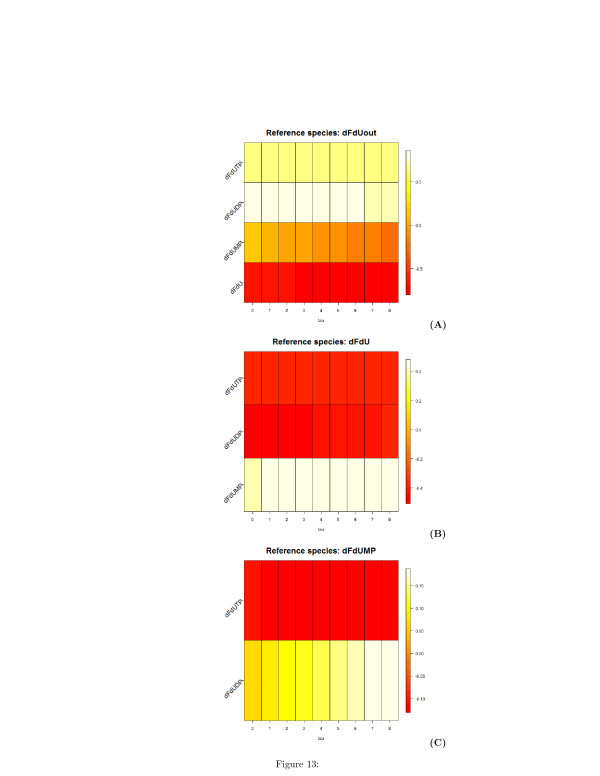
**Heatmap representation of the time-lagged correlations between species obtained from synthetic time series of metabolites concentration.(A)** Reference species is the intra-cellular of dFdU. **(B)** Reference species is the intracellular dFdU. **(C)** Reference species is the mono-phosphate metabolite of dFdU. On the x-axis, the values of the delay *τ* are reported.

The undirected unsigned graph represented in two and three dimensions are shown in Figures 10 (A) and 10 (B), respectively. Similarly to Table 1, Tables 2 and 3 list the time lags corresponding to the minimum and the maximum of the correlation **C**_*ij*_(*τ*), and Figures 11, 12, and 13 show the heatmap representation of the behavior of the correlation as function of *τ*. A monotonic behavior of the correlation vs the time lag is observed for almost all the pairs of the species. This indicates again the presence of chains of reversible reactions involving the species. An elementary single reaction is inferred whenever the maximum correlation between the reference species and another potentially interacting species occurs at a time lag *τ*^*′*^ equal to the average value of the *τ*s at which a given species exhibits the maximum correlation with the other. In this experiment *τ*^*′*^=7.27.

**Table 2:** **Minimum and maximum correlation and corresponding value of the lag*τ*(*τ***_***min_corr***_**and*τ***_***max_corr***_**, respectively) obtained from synthetic time series of dFdC and its metabolites**

**Reference species**	**Species**	***τ***_***max_corr***_	**Max correlation**	***τ***_***min_corr***_	**Min correlation**
dFdCout	dFdCout	0	1	8	0.0002199688
dFdCout	dFdC	2	0.523715	0	0.1466252
dFdCout	dFdCMP	7	0.3980003	0	-0.0301777
dFdCout	dFdCDP	8	-0.1001522	0	-0.3789898
dFdCout	dFdCTP	8	-0.2498713	0	-0.3428283
dFdCout	dFdUout	0	-0.1964403	8	-0.2029342
dFdCout	dFdU	8	0.1920973	1	-0.2593897
dFdCout	dFdUMP	8	-0.2603844	0	-0.4254257
dFdCout	dFdUDP	0	-0.2085703	8	-0.2154594
dFdCout	dFdUTP	0	-0.1226727	8	-0.126728
dFdC	dFdC	0	1	8	0.4815612
dFdC	dFdCMP	3	0.9411531	8	0.7860167
dFdC	dFdCDP	8	0.1379433	0	-0.3858859
dFdC	dFdCTP	8	-0.2253363	0	-0.6472458
dFdC	dFdUout	0	-0.6342664	8	-0.6438545
dFdC	dFdU	6	0.6141992	0	0.0142063
dFdC	dFdUMP	8	-0.1229368	0	-0.6612913
dFdC	dFdUDP	8	-0.6378404	0	-0.6527617
dFdC	dFdUTP	0	-0.4012575	8	-0.4125833
dFdCMP	dFdCMP	0	1	8	0.6754058
dFdCMP	dFdCDP	8	0.3596562	0	-0.01262979
dFdCMP	dFdCTP	8	0.0264333	0	-0.403415
dFdCMP	dFdUout	8	-0.750782	0	-0.7628276
dFdCMP	dFdU	5	0.7362333	0	0.4506942
dFdCMP	dFdUMP	8	0.09732282	0	-0.3657606
dFdCMP	dFdUDP	8	-0.7043206	0	-0.7583209
dFdCMP	dFdUTP	0	-0.5428359	8	-0.5446803
dFdCDP	dFdCDP	0	1	8	0.6404886
dFdCDP	dFdCTP	0	0.9100672	8	0.7518772
dFdCDP	dFdUout	8	0.01484932	0	-0.1177313
dFdCDP	dFdU	0	0.5647046	8	-0.008875956
dFdCDP	dFdUMP	0	0.8015955	8	0.5253429
dFdCDP	dFdUDP	8	0.07034648	0	-0.05754906
dFdCDP	dFdUTP	8	-0.521363	0	-0.5894032
dFdCTP	dFdCTP	0	1	8	0.6794037
dFdCTP	dFdUout	8	0.3093714	0	0.1864065
dFdCTP	dFdU	0	0.3050905	8	-0.2878365
dFdCTP	dFdUMP	0	0.8551774	8	0.4323933
dFdCTP	dFdUDP	8	0.3505927	0	0.2575427
dFdCTP	dFdUTP	8	-0.2830911	0	-0.3565221

**Table 3:** **Minimum and maximum correlation and corresponding value of the lag*τ*(*τ***_***min_corr***_**and*τ***_***max_corr***_**, respectively) obtained from the synthetic time series of dFdU metabolites**

**Reference species**	**Species**	***τ***_***max_corr***_	**Max correlation**	***τ***_***min_corr***_	**Min correlation**
dFdUout	dFdUout	0	1	8	0.8183285
dFdUout	dFdU	0	-0.6570017	7	-0.7962606
dFdUout	dFdUMP	0	0.1308286	8	-0.2427138
dFdUout	dFdUDP	0	0.8595276	8	0.7535818
dFdUout	dFdUTP	8	0.6199872	0	0.6165086
dFdU	dFdU	0	1	8	0.4509447
dFdU	dFdUMP	3	0.4809823	0	0.4264742
dFdU	dFdUDP	8	-0.3964367	0	-0.506585
dFdU	dFdUTP	8	-0.400108	0	-0.4027564
dFdUMP	dFdUMP	0	1	8	0.5456419
dFdUMP	dFdUDP	8	0.1875394	0	0.06438349
dFdUMP	dFdUTP	0	-0.1140161	8	-0.1304115
dFdUDP	dFdUDP	0	1	8	0.8698683
dFdUDP	dFdUTP	8	0.567206	0	0.5078434
dFdUTP	dFdUTP	0	1	8	0.9067803

For more clarity, we did not indicate the directions of the edges on the graph in Figures 10 (A) and 10 (B), but we show the directions of each inferred interaction in Table 4. The third column of the table tags with the letter “E” the expected reactions (i.e. the reactions depicted in the cartoon of Figure 3), and with the letter “U” those unexpected and incorrect, and with the letter “P” those reactions that are unexpected as direct reactions but that are plausible. In fact, two species could appear to be significantly correlated (i.e. connected by an edge) even if their interaction is mediated by intermediate biotransformations.

**Table 4:** Set of reactions inferred by the time-lagged correlation inference method from synthetic time series data: we denote with the letter “E” the expected reactions, and with a letter “U” those unexpected and incorrect, and with the letter P those reactions that are unexpected but that can be easily explained and are plausible in terms of correlation; the reaction dFdCMP → dFdU (i.e. reaction E13 in Table 6) has been not detected

**Reaction ID**	**Reaction**	**Rate constant (*k ± Δk*)**	**Expected/Unexpected**
R1	dFdCout $\overset{k_{1}}{\to }$ dFdC	7.10898534 ± 0.08340605	E
R2	dFdC $\overset{k_{2}}{\to }$ dFdCout	0.77373197 ± 0.20074963	E
R3	dFdCout $\overset{k_{3}}{\to }$ dFdCMP	0.70443170 ± 0.21974489	P
R4	dFdCMP $\overset{k_{4}}{\to }$ dFdCout	2.97694326 ± 1.04080703	P
R5	dFdC $\overset{k_{5}}{\to }$ dFdCMP	0.42700844 ± 0.15141087	E
R6	dFdCMP $\overset{k_{6}}{\to }$ dFdC	1.05564461 ± 0.29004594	E
R7	dFdCDP $\overset{k_{7}}{\to }$ dFdCTP	0.67814401 ± 0.33891991	E
R8	dFdCTP $\overset{k_{8}}{\to }$ dFdCDP	0.24600288 ± 0.12911296	E
R9	dFdC $\overset{k_{9}}{\to }$ dFdUout	0.07429936 ± 0.10274560	P
R10	dFdUout $\overset{k_{10}}{\to }$ dFdC	1.84455256 ± 0.41277353	U
R11	dFdC $\overset{k_{11}}{\to }$ dFdU	0.05303525 ± 0.11826494	E
R12	dFdU $\overset{k_{12}}{\to }$ dFdC	0.42656017 ± 0.36820500	U
R13	dFdCMP $\overset{k_{13}}{\to }$ dFdU	0.01746121 ± 0.04239150	U
R14	dFdU $\overset{k_{14}}{\to }$ dFdCMP	1.15296892 ± 0.87614147	U
R15	dFdCDP $\overset{k_{15}}{\to }$ dFdU	0.74470692 ± 0.04372718	U
R16	dFdU $\overset{k_{16}}{\to }$ dFdCDP	0.13066227 ± 0.30402961	U
R17	dFdCTP $\overset{k_{17}}{\to }$ dFdU	0.21526282 ± 0.24385105	U
R18	dFdU $\overset{k_{18}}{\to }$ dFdCTP	0.22459952 ± 0.31608938	U
R19	dFdUout $\overset{k_{19}}{\to }$ dFdU	0.15238122 ± 0.22702423	E
R20	dFdU $\overset{k_{20}}{\to }$ dFdUout	0.90365762 ± 1.15063193	E
R21	dFdCDP $\overset{k_{21}}{\to }$ dFdUMP	0.33079357 ± 0.29670272	U
R22	dFdUMP $\overset{k_{22}}{\to }$ dFdCDP	0.14176496 ± 0.32573757	U
R23	dFdCTP $\overset{k_{23}}{\to }$ dFdUMP	0.03484985 ± 0.08815358	U
R24	dFdUMP $\overset{k_{24}}{\to }$ dFdCTP	0.23689470 ± 0.32365144	U
R25	dFdU $\overset{k_{25}}{\to }$ dFdUMP	1.30633488 ± 0.31031032	E
R26	dFdUMP $\overset{k_{26}}{\to }$ dFdU	1.10583395 ± 0.20384256	E
R27	dFdUout $\overset{k_{27}}{\to }$ dFdUDP	0.19345312 ± 1.03917708	P
R28	dFdUDP $\overset{k_{28}}{\to }$ dFdUout	0.74875449 ± 1.17435092	P
R29	dFdC $\overset{k_{29}}{\to }$ dFdUTP	0.12752520 ± 0.13267091	U
R30	dFdUTP $\overset{k_{30}}{\to }$ dFdC	0.99825505 ± 0.40035050	U
R31	dFdCMP $\overset{k_{31}}{\to }$ dFdUTP	0.52124642 ± 0.95261538	U
R32	dFdUTP $\overset{k_{32}}{\to }$ dFdCMP	0.46822249 ± 0.95099997	U

The rate constants have been estimated with KInfer. Theirs values are reported with a precision of 8 digits. The third column of the table denote with the letter “E” the expected reactions, and with a letter “U” those unexpected and incorrect, and with the letter “P” those reactions that are unexpected but that can be easily explained and are plausible in terms of correlation. The reaction *dFdCMP*→*dFdU* (i.e. reaction E13 in Table 6) has been not detected.

**Table 5:** The set of reactions for which the ratio between the estimate of the rate constant and the estimate of its error is equal or greater than one

**Reaction ID**	**Rate constant**	**Expected/Unexpected**
R1	*k*_1_=7.11±0.08	E
R2	*k*_2_=0.8±0.2	E
**R3**	*k*_3_=0.7±0.2	P
**R4**	*k*_4_=3±1	P
R5	*k*_5_=0.43±0.15	E
R6	*k*_6_=1.1±0.3	E
R7	*k*_7_=0.7±0.3	E
R8	*k*_8_=0.25±0.13	E
**R10**	*k*_10_=1.8±0.4	U
R14	*k*_14_=1.2±0.9	E
**R15**	*k*_15_=0.74±0.04	P
R25	*k*_25_=1.3±0.3	E
R26	*k*_26_=1.1±0.2	E
**R30**	*k*_30_=1.0±0.4	U

The reactions R11, R19 and R20 tagged as “expected” by the correlation-based inference procedure and reported in Table 4 have been cut off by the network calibration procedure, and thus they do not appear in this Table. The reactions whose ID is bold faced are discussed in the text in Section “Results”.

The comparison of this set of reactions with those observed in experiments and depicted in Figure 3 reveals the presence of false positive in the inferred reactions set. The calibration of the inferred model with KInfer allowed to detect the null kinetics, i.e. those reactions whose rate constant *k* is null or it is affected by an error *Δk* equal or greater than the estimated value. The cases in which the ratio between the estimate of the parameter value and the estimate of its error is equal or greater than one, are interpreted as noise and not as a biochemical kinetics governing the time behavior of the species concentration. The calibration of this inferred model with KInfer gives as non-null kinetics the reaction showed in Table 5. In this set of reactions, R3, R4, R10 and R15 are also included, although in these reactions the interaction between the reactants are mediated by intermediate reactions. Reaction R30 has been inferred, but it is not expected at all.

**Table 6:** The set of expected real reactions (from the cartoon of Figure 3)

**Reaction ID**	**Reaction**
E1	dFdCout → dFdUout
E2	dFdCout → dFdC
E3	dFdC → dFdCout
E4	dFdC → dFdU
E5	dFdU → dFdUout
E6	dFdUout → dFdU
E7	dFdC → dFdCMP
E8	dFdCMP → dFdC
E9	dFdCMP → dFdCDP
E10	dFdCDP → dFdCMP
E11	dFdCDP → dFdCTP
E12	dFdCTP → dFdCDP
E13	dFdCMP → dFdUMP
E14	dFdU → dFdUMP
E15	dFdUMP → dFdU
E16	dFdUMP → dFdUDP
E17	dFdUDP → dFdUMP
E18	dFdUDP → dFdUTP
E19	dFdUTP → dFdUDP

According to reaction R3, the decrement of dFdCout is directly proportional to the increment of dFdCMP via a single reaction consuming dFdCout and producing dFdCMP. However, the model includes also the uptake of external dFdC into the cell and its excretion, i.e. $\mathrm{dFdCout}\;\overset{k_{1}}{\underset{k_{2}}{⇌}}\;\mathrm{dFdC}$. Once gemcitabine is inside the cell, it undergoes a reversible reaction of phosphorilation, i.e: $\mathrm{dFdC}\;\overset{k_{5}}{\underset{k_{6}}{⇌}}\;\text{dFdCMP}$. The estimates of the rates obtained with KInfer for these reactions are: *k*_1_=7.11, *k*_2_=0.8, *k*_5_=0.43 and *k*_6_=1.1. *k*_1_ is one order of magnitude greater than *k*_5_, and similarly *k*_6_ is one order of magnitude greater than *k*_5_. As consequence, since (i) the uptake of dFdCout is much faster than the phosphorilation reaction, and (ii) the dephosphorilation of dFdC is much faster than the excretion from the cell [7,8], the correlation-based inference model detects a significant correlation between dFdCout and dFdCMP through the reactions R3 and R4. Nevertheless, the algorithm is able to detect the uptake and the excretion of the dFdC (Reactions R1 and R2).

**Figure 14:**
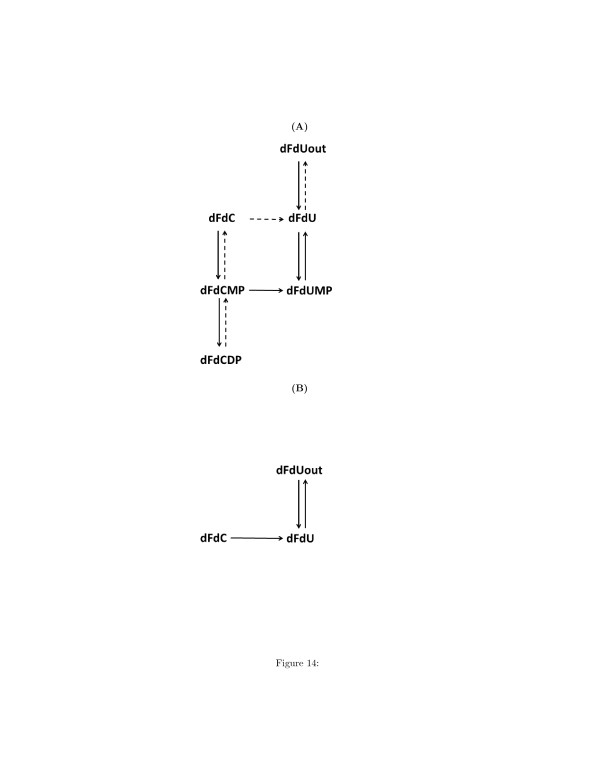
**(A) Pathway connecting dFdCDP and the intra-cellular concentration of dFdU (dotted arrows).(B)** The interaction between dFdUout and dFdC is mediated by the pathway illustrated in this figure. Therefore the reaction R10 (see Table 5) is a false positive inferred reaction.

Similarly, the algorithm infers also reaction R15: $\text{dFdCDP}\overset{k_{15}}{\to }\mathrm{dFdU}$. dFdCDP and dFdU are connected through a pathway depicted in Figure 14 (A) (dotted arrows). The variation of the concentration of dFdU is correlated to the variation of the concentration of dFdC and dFdUMP. In turn, the variation of the concentration of dFdC and dFdUMP are both correlated to the variation of the concentration of dFdCMP.

Finally, the correlation-basednetwork inference algorithm does not detect the reaction *dFdCMP*→*dFdU* that is reported in the cartoon of Figure 3, and later in the Table 6 (i.e reaction E13).

Table 5 reports also Reaction R10: $\text{dFdUout}\overset{k_{10}}{\to }\text{dFdC}$. According to the current knowledge, the interaction between dFdUout and dFdC is mediated by the pathway illustrated in Figure 14 (B). Therefore reaction R10 is not correct, because a decrement of the extra-cellular concentration of dFdU does not cause an increment of the intracellular concentration of dFdC. The opposite is plausible: a decrement of the intracellular dFdC is proportional to an increment of the intracellular dFdU, that can be released outside the cell. Finally, Table 5 reports reaction R30, that is not correct, because, as we can see in Figure 3, the metabolites of phosphorylated metabolites of dFdU are not converted in any metabolites of dFdC.

The reactions R11, R19 and R20 tagged as “expected” by the correlation-based inference procedure and reported in Table 4 have been cut off by the network calibration procedure, and thus they do not appear in Table 5.

Summarizing, from the model in Figure 3 and the list in Table 6, we see that 19 reactions are expected. The network inference algorithm paired to the parameter inference algorithm of KInfer correctly inferred 12 reactions. It inferred two false positive, i.e. R10 and R30. Moreover, it missed Reactions R19 and R20: they are expected, but they have been not detected. We introduce two parameters to evaluate the performance of the procedure: the sensitivity and the accuracy. The sensitivity is defined by the ratio between the number of detected edges and the number of expected edges. The accuracy is defined as the ratio between the number of correctly detected edges and the number of detected edges. The number of expected edges is the numer of expected reactions, that is 19 (see Table 6). According to these definitions we have

$$\begin{array}{l} \;\;\;\;\text{sensitivity}\;=\;\frac{\text{Nr. of detected edges(the “plausibles” included)}}{\text{Nr. expected edges}}\\ \;=\frac{12}{19}=63,2\% \end{array}$$

$$\begin{array}{l} \;\;\;\;\text{sensitivity}\;=\;\;\frac{\text{Nr. of detected edges(the “plausibles” excluded)}}{\text{Nr. expected edges}}\\ \;=\frac{9}{19}=47,3\%\\ \;\text{accuracy}=\frac{\text{Nr. of correctly detected}}{\text{Nr. detected edges}}=\frac{9}{12}=75\mathrm{\%.} \end{array}$$

Our procedure on the case study of gemcitabine metabolism outperforms the algorithm in [44] that has been designed for the determination of chemical reaction network interconnections from time series data too. However, we point out that the sensitivity and the accuracy defined in this way are global measures that average over the whole network giving two values as representative of the performance of the inference algorithm. A better assessment of the inference algorithm could be conducted if we disposed either of real biological but small-scale data. A comparison of these performances with the performances of other state-of-art tools is still in progress. Preliminary results of this analysis show us that the outcome of the network inference varies between tools and can be complementary. Moreover, each tool is tailored to a specific biological pathway and experimental set up. For this reason mainly, in the scientific community there is no general consensus that would have been reached declaring one network inference method as gold standard [45].

## Conclusions

We presented a method for the prediction of the interactions within reaction networks from experimental time series of the concentration of the species composing the system. The overall aim of our investigation was to develop a procedure of system identification in which the parameter inference acts as tool of model refinement and elimination of false positive interconnections. Both the network topology inference and the parameter estimation have been designed to predict reaction pathways in chemical kinetic systems from noisy time resolved concentration measurements.

We developed a method that does not require any a priori information about the topology of the interconnections. It consists of two parts: the estimation of time-lagged correlation function between the components of the systems for the determination of direct edges between the components and the inference of the kinetic parameters to establish real dynamics and to cut “null dynamics” edges instead. The method has been developed to deducing the connectivities of chemical species, the reaction pathway, and the reaction mechanism of complex reaction systems. The procedure can process more than 3,000 reactions involving hundreds of species. In particular, our methodology coupling the connectivities inference to the parameter inference reveals to be suitable to infer reactions mechanisms of complex systems as drug metabolic network, where many feedback loops and reversible reactions are present. The data driven estimation of the time lag interval facilitates the use of the tool and minimize the a priori information to input. Good performances have been obtained on the test case of gemcitabine metabolism, that is supported by recent experimental studies. Finally, this work highlights five main aspects determining a sensitive and accurate inferability of a biochemical network with time-lagged correlation-based methods: (i) the availability of replicates of experiments, so that averaged time series can be used to soften the noise in the input data; (ii) the availability of data having a time resolution suitable to capture the time of onset of the correlation between the species and the number of chemical species that can be measured simultaneously; (iii) the availability of local measures on the level of individual edges and subnetworks, instead of having only global measures of time series averaging over the whole network dynamics [45]; (iv) the effects of measurement error, and (v) the presence of unmeasured species. These issues have to be addressed before adopting inference methods in the daily laboratory practice for any specific problem.

## Competing interests

The authors declare that they have no competing interests.

## Authors’ contributions

Each author contributed to this work in compliance with his/her expertise field. PL developed the mathematical framework of the network inference methods and contributed to the development of the software tool implementing it. PL also designed the in silico experiments and the tests of the method on the case study of gemcitabine metabolism. DM contributed to the analysis and validation of the results of the inference. GF performed the experimental data preprocessing necessary to use them as inputs of the inference procedure. CP and AC dealt with the program coding the mathematical expressions and the data structures of the inference method to allow its execution on computer. They also contributed to testing and validating the theory, and provided solutions for an efficient implementation. All authors read and approved the final manuscript.
